# Cytomegalovirus in urinary sediment in patients with acute kidney injury

**DOI:** 10.1186/s12882-021-02377-z

**Published:** 2021-05-08

**Authors:** Sahra Pajenda, Sebastian Kapps, Daniela Gerges, Gregor Hoermann, Ludwig Wagner, Nina Buchtele, Barbara Geist, Robert Strassl, Alice Schmidt, Wolfgang Winnicki

**Affiliations:** 1 Department of Internal Medicine III, Division of Nephrology and Dialysis, Medical University of Vienna, Waehringer Guertel 18-20, 1090 Vienna, Austria; 2 Department of Laboratory Medicine, Medical University of Vienna, Vienna, Austria; 3 MLL Munich Leukemia Laboratory, Munich, Germany; 4 Department of Internal Medicine I, Stem Cell Transplantation Unit, Medical University of Vienna, Vienna, Austria; 5 Department of Biomedical Imaging and Image- Guided Therapy, Division of Nuclear Medicine, Medical University of Vienna, Vienna, Austria; 6 Department of Laboratory Medicine, Division of Clinical Virology, Medical University of Vienna, Vienna, Austria

**Keywords:** Acute kidney injury, Cytomegalovirus, Immunosuppression, Renal transplantation, Urinary sediment

## Abstract

**Background:**

Immunosuppression in solid organ transplantation is associated with frequent infections. Renal allograft recipients are susceptible to opportunistic infections and can acquire human cytomegalovirus (HCMV) infections even within the allograft. There, HCMV can be found in both the glomerulus and tubular cells, but is mostly restricted to specific and circumscribed sites. Therefore, not all organ infections are identifiable by immunohistology for HCMV proteins in fine needle core biopsies. Thus, we performed a urinalysis study to search for HCMV-specific RNA transcripts in the urine sediment of patients with acute kidney injury.

**Methods:**

Urinary sediment of 90 patients with acute kidney injury (AKI), including 48 renal transplant recipients (RTX) and 42 non-transplant recipients (nRTX), was collected from morning urine for RNA extraction and reverse transcription. The copy number of HCMV transcripts was evaluated using a UL132 HCMV-specific probe set and by real-time quantitative polymerase chain reaction (RT-qPCR).

**Results:**

Of the 48 RTX patients, ten showed HCMV copies in their urine sediment cells. Within this group, three recipients had negative HCMV serology and received an allograft from an HCMV-seropositive donor. In addition, all three RTX patients on a belatacept-based immunosuppressive regimen had HCMV transcripts in their urine. Of the 42 nRTX patients, only two had detectable HCMV transcripts in urine sediment cells and both were under immunosuppression.

**Conclusions:**

Ten immunosuppressed renal allograft recipients and two immunosuppressed non-transplant patients with AKI showed HCMV copies in urine sediment. Thus, HCMV positivity in urinary sediment appears to be associated with immunosuppression. This study describes a novel noninvasive method for detection of HCMV in urinary sediment. Whether all HCMV infections can be detected or only those with viral replication warrants further investigation.

## Background

In comparison to healthy individuals, renal transplant (RTX) recipients treated with immunosuppressive agents are more likely to develop HCMV viremia and infections. In particular, HCMV seronegative recipients (R-) from seropositive donors (D+) are at increased risk to develop a novel HCMV infection [1, 2]. Cell-mediated and humoral immune defense are markedly compromised in transplant recipients on immunosuppressive therapy with calcineurin inhibitors or belatacept [3, 4], among others, making the renal allograft a target for infection with epitheliotropic HCMV [5]. Of note, allograft rejection has been reportedly associated with HCMV infections [5] and leads to increased risk for death-consorted graft failure in transplant recipients [6].

Kidney biopsy is a potent diagnostic measure in acute kidney injury (AKI) when, e.g., allograft rejection must be ruled out. However, histopathology in general is rather limited in the diagnosis of HCMV infection, although various antibodies can detect viral proteins in infected cells [7]. HCMV infection is variably distributed throughout organs and may be overlooked in biopsy specimens. Therefore, staining for early HCMV antigen is not part of the scope of conventional transplant histopathology. Quantitative titer increase of HCMV in blood does not correlate with HCMV infection in renal allografts; nevertheless, both renal tubule epithelia [8] and cells at the glomerulus [9–11] are targets of HCMV infection, leading to cellular destruction and their excretion in urine [12, 13].

Despite routine HCMV-specific treatment, HCMV-seronegative recipients (R-) of HCMV-seropositive donors (D+) bear an independent risk for graft failure, all-cause mortality and infection-related mortality, as reported in a recent study including data from the United Network for Organ Sharing/Organ Procurement and Transplantation Network [6].

In this retrospective work, we analyzed the urine sediment of transplant and non-transplant patients with acute kidney injury for HCMV transcripts. Urinary sediment was collected and total RNA was extracted, reverse transcribed and tested for the presence of HCMV-UL132 transcript numbers by real-time quantitative polymerase chain reaction (RT-qPCR). Furthermore, immunofluorescence staining of urine sediment for HCMV- immediate early antigen (HCMV-IEA) was performed. The medical history of all patients was followed 24 months before the onset and 8 months after the episode of acute kidney injury.

## Material & methods

### Study population

Patients undergoing AKI stages I, II or III according to the KDIGO definition [14] were consecutively enrolled from 2016 to 2020 at the Department of Medicine III, Division of Nephrology and Dialysis at the Medical University of Vienna. Inclusion criteria were an age above 18 years and the presence of acute kidney injury. The main exclusion criteria were patients with end stage renal disease receiving renal replacement therapy. Written informed consent was obtained from all study participants. The study was approved by the Ethics committee of the Medical University of Vienna (EK 1043/2016).

 All methods were performed in accordance with relevant guidelines and regulations.

### Patient records and clinical data

Patient data comprising demographics, comorbidities, laboratory parameters including renal function parameters and HCMV status were extracted from the medical database of the Medical University of Vienna.

### Urine collection and reverse transcription

Seven ml of morning urine were collected and immediately centrifuged at 3500 revolutions per minute (RPM). The resultant sediment was lysed in 1000 µl TriFast (Peqlab, 30-2010) and frozen at -20 °C. For isolation of total RNA the cell lysate was thawed and left for 5 min at room temperature. Following mixing the cell lysate with 300 µl of chloroform and centrifugation for 10 min at 12 000 g, the RNA containing supernatant was taken off and RNA was precipitated using 250 µl isopropanol. The washed RNA pellet was re-dissolved in RNase free water and subjected to reverse transcription. In brief: 400 ng of total RNA was mixed with random primers (Invitrogen, 48190-011), dNTPs heated for 3 min to linearize RNA and rapidly chilled in ice water while Superscript® III (Invitrogen, 180808-044) was added together with dithiothreitol (DTT) and reverse transcription buffer. The reaction was incubated at 25 °C for 5 min for primer annealing and synthesis was carried out at 52 °C for further 30 min. The enzyme function was stopped by heating the reaction at 75 °C for 10 min.

### RT-qPCR of urinary cell cDNA and HCMV copy number determination

An HCMV UL132 TaqMan probe (Applied Biosystems, Pa03453400) was cloned into TOPO cloning vector and One Shot (Invitrogen, C4040-10) chemically competent cells were transformed with the resultant plasmid using the heat shock method. In brief: two µl of PCR product was incubated with TOPO-vector for 5 min at room temperature. One µl of the plasmid was combined with 50 µl One Shot E.coli and a 45 s heat shock was carried out in a water bath at 42 °C. Cells were briefly chilled on ice and incubated for one hour at 37 °C in 250 µl SOC medium. Spreading of E.coli was performed on LB/Amp plates and individual clones were collected 14 h later. The UL132 encoding plasmid was isolated and has been further used as positive control and UL132 copy number determination by including a 10- fold dilution standard series in RT-qPCR experiments as follows.

One µl of urinary cell cDNA or 10-fold dilution series of UL132 encoding plasmid was mixed with 1 µl of UL132-specific TaqMan probe and 5 µl universal mastermix (Applied Biosystems, 4,304,437) and diluted to a final volume of 10 µl, all in duplicate. The sample set up was transferred into a 96 well PCR plate and inserted into the ONEstep RT-qPCR machine which was set for recording 46 PCR cycles. For copy number evaluation the 10-fold serial dilution of a plasmid encoding the UL132 transcript was used. This reflected a spectrum of 0.5 × 10^2^-10 × 10^6^ UL132 copies as standard curve.

### Cytospin preparation for urine sediment

Seven ml urine was centrifuged at 3500 RPM for 10 min. The resultant pellet was re-suspended in 1500 µl tissue culture medium (RPM 1640 containing 10 % new born calf serum). Hundred fifty µl were placed into the funnel of a cyto-centrifuge (Cytospin 3, Shandon, England). Loaded samples were spun at 1200 RPM for 4 min. The resultant cytoslides were air dried for 2 h and either processed immediately or wrapped in aluminum foil and kept at -20 °C for further use.

### Immunofluorescence staining

The cytoslides were fixed in acetone for 5 min. Subsequently, a liquid repellent barrier was drawn using an Aqua-Hold Pap Pen, where urinary cells had been deposited by the cyto-centrifuge.

The mouse monoclonal anti-human cytomegalovirus immediate early antigen (HCMV-IEA) antibody (ARGEGE, Ref 11 − 003, France) was diluted 1:300 in PBS (phosphate buffered saline) with blocking solution (BSA, bovine serum albumin). The rabbit anti-human AQP1 was diluted 1:800 (Millipore AB 2219). Slide incubation for the primary antibody was carried out at 4˚C in a moist chamber and the next day with Alexa Fluor 488 goat anti-rabbit (diluted 1:400) and TRIC goat anti-mouse (diluted 1:400) for 1 h at room temperature. Five minutes before washing 40 µl of DAPI solution was added onto the slide for nuclear counterstain. After each antibody incubation, the slides were washed under constant stirring of the liquid in PBS. Finally, slides were mounted in Vectashield mounting medium for immunofluorescence (Vecotor Laboratories, Burlingham CA), covered with a coverslip and imaged with a Zeiss Axiovert confocal microscope and further processed by Adobe Photoshop version 6.

###  Human cytomegalovirus (HCMV) detection in plasma

Plasma samples were analyzed for the presence of HCMV DNA at the Department of Laboratory Medicine, Division of Clinical Virology at the Medical University of Vienna. Quantitative measurement of HCMV DNA viral load by PCR was performed on an Abbott m2000 platform (Abbott Molecular, Des Plaines, Illinois, USA) using the Abbott Real-Time HCMV assay (limit of detection: 20 copies/ml).

### Statistical analysis

Adherence to a Gaussian distribution was determined using the Kolmogorov-Smirnov test. Normally distributed data were described as means ± SDs, and the independent samples Student t test was utilized to compare continuous variables between the two groups (urinary HCMV transcript positive and negative). In case of a skewed distribution, data were described as medians with interquartile ranges (IQR) and were compared using the Mann-Whitney U test. Qualitative variables were described with counts and percentages and group differences were assessed using contingency tables and the Fisher’s exact test. Data were analyzed with Graphpad Prism (Version 9.0 for Windows). All *P*- values result from 2-sided tests, with significance inferred at *P* < 0.05.

## Results

Urine samples were collected from a total of 90 individuals during their hospitalization for acute kidney injury, obtained on the first day of admission representing the climax of disease. Out of these, 48 subjects were renal transplant recipients, 42 were non-transplant patients. Baseline characteristics of the study population are given in Table 1.

**Table 1:** Baseline Characteristics of all Study Patients. *AKI* acute kidney injury, *CKD* chronic kidney disease, *DGF* delayed graft function, *HCMV* human cytomegalovirus, *IRI* ischemia reperfusion injury, *n.a.*not applicable, *RTX* renal transplantation

	Study Subjects total	RTX Patients	Non-RTX Patients
**Number of subjects**	90	48	42
**Age (years)**	58.46 ± 15.96	55.81 ± 12.95	61.48 ± 18.37
**Gender - number (%)**			
**Male sex**	61 (67.78)	33 (68.75)	28 (66.67)
**Female sex**	29 (32.22)	15 (31.25)	14 (33.33)
**Caucasian - number (%)**	89 (98.89)	48 (100.00)	41 (97.62)
**Treated with immunosuppression - number (%)**	52 (57.78)	48 (100.00)	5 (11.90)
**Comorbidities - number (%)**			
**Hypertension**	75 (83.33)	42 (87.50)	33 (78.57)
**Diabetes mellitus**	26 (28.89)	17 (35.42)	9 (21.43)
**Cardiovascular disease**	27 (30.00)	17 (35.42)	10 (23.81)
**Atrial fibrillation**	22 (24.44)	10 (20.83)	12 (28.57)
**Cerebrovascular disease**	12 (13.33)	8 (16.67)	4 (09.52)
**Peripheral artery disease**	13 (14.44)	12 (25.00)	1 (02.38)
**Chronic kidney disease - number (%)**			
**CKD Stage 1**	16 (17.78)	0 (0.00)	16 (38.10)
**CKD Stage 2**	10 (11.11)	1 (02.08)	9 (21.43)
**CKD Stage 3**	19 (21.11)	8 (16.67)	11 (26.19)
**CKD Stage 4**	7 (07.78)	5 (10.42)	2 (04.76)
**CKD Stage 5**	12 (13.33)	8 (16.67)	4 (09.52)
**Post-transplant phase**	26 (28.89)	26 (54.17)	0 (0.00)
**Acute kidney injury - number (%)**			
**AKI Stage 1**	10 (11.11)	7 (14.58)	3 (07.14)
**AKI Stage 2**	6 (06.67)	2 (04.17)	4 (09.52)
**AKI Stage 3**	48 (53.33)	13 (27.08)	35 (83.33)
**DGF/IRI**	26 (28.89)	26 (54.17)	0 (0.00)
**HCMV detection in urinary sediment - number (%)**	12 (13.33)	10 (20.83)	2 (04.76)
**HCMV detection in plasma - number (%)**	3 (03.33)	3 (06.25)	0 (0.00)

Stages of AKI were assessed by serum creatinine levels; furthermore, for all patients the underlying stage of chronic kidney disease (CKD) was reported according to current guidelines [15, 16]. As shown in Table 1 most patients from both transplant and non-transplant groups presented with AKI stage 3 (RTX: *n* = 13; nRTX: *n* = 35). The most common causes of acute kidney injury were infection and sepsis-related (*n* = 24) and prerenal (*n* = 14). Six patients suffered from AKI due to shock, in 9 patients AKI occurred due to a renal cause. Other reasons for AKI were postrenal (*n* = 4), toxic (*n* = 4), acute graft rejection (*n* = 2) and trauma-related (*n* = 1). Furthermore, twenty-six transplant recipients were assigned in the postoperative period of renal transplantation with no or deteriorating renal function. Among these, reasons for renal malfunction were allograft rejection (*n* = 9), thrombotic microangiopathy (*n* = 1) and tubular damage (*n* = 3). In 6 patients, histology of the graft biopsy revealed no apparent reason for delayed graft function (DGF), and in 7 patients, graft biopsy was not performed owing to incipient improvement of renal function or patient refusal.

The mean age of RTX patients was 55.81 ± 12.95 years and 61.48 ± 18.37 years of nRTX patients. All renal transplant patients received immunosuppressive therapy, most of them being on a calcineurin inhibitor-based (*n* = 43), belatacept-based (*n* = 3) or other regimen (*n* = 2) (Table 2). The average cold ischemia time was 14.88 ± 5.62 h and donor age was 56.00 ± 16.45 years.

**Table 2:** Clinical Characteristics and Donor-specific Data according to HCMV status in Urinary Sediment of RTX Patients. *ATG* antithymocyte globulin, *CNI* calcineurin inhibitor, *DSA* donor specific antibodies, *D/R* donor/recipient, + positive, - negative, *HCMV* human cytomegalovirus, *IAS* immunoadsorption, *IQR* interquartile range, *n.a.* not applicable, *RTX* renal transplantation

	HCMV positive	HCMV negative	***P***-value
**Number of subjects**	10	38	
**Age (years)**	56.1 ± 13.43	55.74 ± 12.82	0.938
**Gender - number (%)**			
**Male sex**	7 (70.00)	26 (68.42)	1.000
**Female sex**	3 (30.00)	12 (31.58)	1.000
**Donor specific data**			
**Donor Age (years)**	58.20 ± 17.33	55.44 ± 16.14	0.638
**Cold Ischemia Time (hours)**	17.56 ± 4.03	13.28 ± 5.68	0.031
**Deceased Donor - number (%)**	10 (100.00)	32 (84.21)	0.320
**Living Donor - number (%)**	0 (0.00)	6 (15.79)	0.320
**Immunosuppression - number (%)**			
**CNI-based Regimen**	7 (70.00)	36 (94.74)	0.054
**Belatacept-based Regimen**	3 (30.00)	0 (0.00)	0.007
**Other**	0 (0.00)	2 (05.26)	1.000
**History of ATG/IAS Induction Therapy**	0 (0.00)	5 (13.16)	0.569
**Rejection Therapy**	2 (20.00)	9 (23.68)	1.000
**Median time (IQR) after transplantation in days**	94 (51 – 596)	17 (8 – 76)	0.019
**Baseline DSA - number (%)**			
**positive**	0 (0.00)	6 (15.79)	0.320
**negative**	10 (100.00)	32 (84.21)	0.320
**Allograft rejection - number (%)**			
**yes**	2 (20.00)	10 (26.32)	1.000
**no**	8 (80.00)	28 (73.68)	1.000
**Baseline HCMV status - number (%)**			
**D-/R-**	0 (0.00)	4 (10.53)	0.567
**D-/R+**	2 (20.00)	13 (34.21)	0.472
**D+/R-**	3 (30.00)	5 (13.16)	0.336
**D+/R+**	5 (50.00)	16 (42.10)	0.729
**HCMV- specific treatment at study timepoint - number (%)**			
**yes**	3 (30.00)	12 (31.58)	1.000
**no**	7 (70.00)	26 (68.42)	1.000
**HCMV infection at study timepoint - number (%)**			
**yes**	1 (10.00)	2 (05.26)	0.512
**no**	9 (90.00)	36 (94.74)	0.512
**History of HCMV infection within the previous 2 years - number (%)**			
**yes**	2 (20.00)	3 (07.89)	0.276
**no**	8 (80.00)	35 (92.11)	0.276
**HCMV infection during 8-months follow up - number (%)**			
**yes**	1 (10.00)	6 (15.79)	1.000
**no**	9 (90.00)	32 (84.21)	1.000
**Median (IQR) of HCMV urine sediment (copies/7mL urine)**	788 (552 – 928)	n.a.	

RNA was extracted from urine sediments and immediately reverse-transcribed. The resulting cDNA was tested for UL132-RNA expression in urinary sediment using a UL132 HCMV-specific probe set and RT-qPCR. Details on transplantation and type of immunosuppression, HCMV serology status, current existing HCMV infection, history of HCMV infection as well as HCMV infection within an 8 months follow-up period of RTX patients with and without HCMV transcripts in the urinary sediment are given in Table 2.

**Fig. 1:**
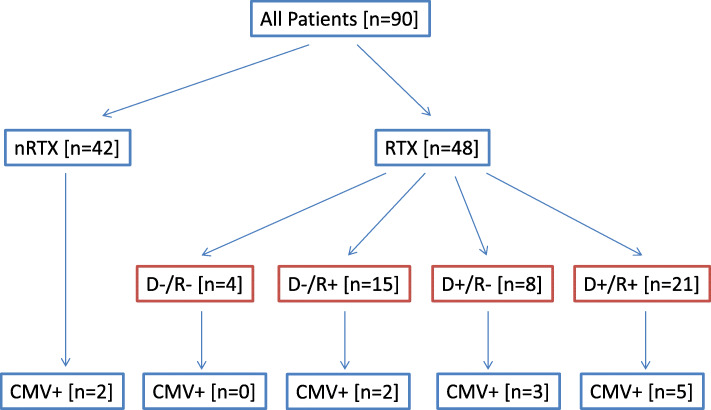
Flowchart of Study Subjects. HCMV transcripts in urine sediment were positive in two of 42 non-transplant patients and in ten of 48 renal transplant recipients. HCMV+, urinary sediment positive for UL132 (HCMV-specific probe set); D/R, donor/recipient; +, positive; -, negative; nRTX, non-transplant recipient; RTX, renal transplant recipient

Ten patients of the renal transplant group tested positive for HCMV transcripts in the urinary sediment. In the non-transplant group, HCMV transcripts were detected in the urinary sediment in only two patients, both under immunosuppression (Fig. 1).

Among renal transplant patients the underlying HCMV IgG status of the donor (D) and recipient (R) at the time of transplantation did not account for the pattern of HCMV detection in urinary sediment (Table 2). Furthermore, no differences in the immunosuppressive regimen, history of induction therapy with antithymocyte globulin / immunoadsorption (ATG/ IAS) and rejection therapy between RTX patients with and without HCMV transcripts in the urinary sediment were found. Of note, patients who tested positive for HCMV transcripts in urine sediment had been under immunosuppressive therapy for a longer time post transplantation compared to patients without HCMV detection (Table 2).

In patients positive for HCMV transcripts in the urinary sediment, the HCMV expression level of the cellular urine sediment, given in copy numbers, varied within a particular range (non- RTX patients: median 772 (IQR, 757–787); all RTX patients: median 788 (IQR, 552–928); D-/R+: median 404 (IQR, 221–587); D+/R-: median 952 (IQR, 750–16,938); D+/R+: median 806 (IQR, 564–857). A urine sediment sample was considered HCMV positive in this analysis with a cycle threshold below 45 cycles.

Out of the ten RTX patients positive for urinary HCMV transcripts, two patients (20 %) had an acute allograft rejection in the early phase around urinalysis, whereas ten of 38 patients (26 %) without urinary HCMV transcripts also experienced graft rejection (Table 2). In the late phase after HCMV transcript detection in urine, no further rejection episodes occurred. In addition, eight out of the ten RTX patients with positive urinary HCMV transcripts had a recovery of kidney function, two patients did not regain renal function, of which one patient died due to severe hemorrhage shock and one patient developed end-stage renal disease. Among all the 90 patients analyzed, three were positive for HCMV DNA in plasma, of which all were RTX patients under immunosuppression. In one of these three patients with high risk HCMV constellation (D+/R-) the urine sediment also proved positive for HCMV UL132 transcript.

**Fig. 2:**
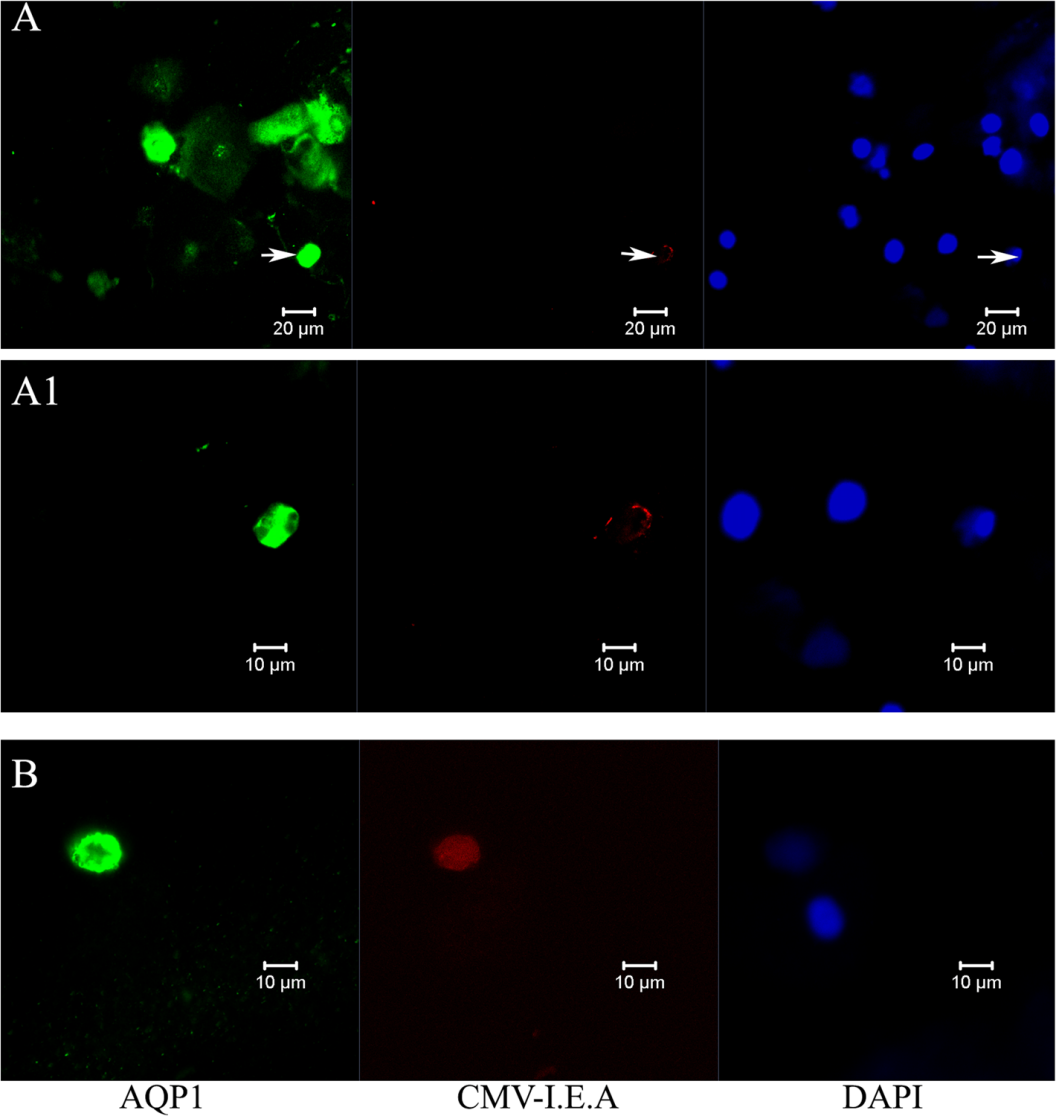
Confocal immunofluorescence staining of urinary sediment for cytomegalovirus immediate early antigen (HCMV-IEA) in aquaporin 1 (AQP1) positive tubular epithelia cells with different distribution pattern. Staining for HCMV-IEA is shown in red, for AQP1 in green and for DAPI in blue. **A**: marginal staining of HCMV-IEA at the nuclear edge. The cell marked with an arrow (→) is shown at higher magnification in **A1**. **B**: HCMV-IEA staining of the nucleus

To further delineate which cell types in the urinary sediment contain HCMV transcripts translated into protein, immunofluorescence staining for detecting the human cytomegalovirus immediate early antigen (HCMV-IEA) was performed. This detected HCMV-IEA expression in AQP1 positive tubular epithelia cells with morphological signs of membrane damage, as shown in Fig. 2.

## Discussion

The goal of this study was to investigate the presence of HCMV infected cells in urine in transplant recipients undergoing AKI. As control, non-transplant patients with AKI were included. The HCMV risk profile of all allograft recipients was assessed, as studies have previously shown that kidneys from HCMV-seropositive donors transplanted into recipients with HCMV-seronegative (D+/R-) profiles were associated with a worse clinical outcome after transplantation [6]. The cohort of patients with AKI and positive urinary HCMV transcripts included predominantly recipients from HCMV-seropositive donors. In addition, all three patients on belatacept-based immunosuppressive regimen were positive for HCMV in urine sediment cells, which is in accordance with data of a recent study [4].

The UL132 used probe set tests for an early response gene which lies in the UL/b’ region and represents a part of the UL146 gene. Although the UL132 contains some polymorphic sites [17] at the 5’ end, it represents an ideal marker for the detection of most HCMV variants using this protein as target. Until now, no data were available on HCMV propagation in renal epithelial cells. Using a Taqman UL132 specific probe and urinary sediment cDNA from 90 patients undergoing AKI, the presence of HCMV transcripts could be analyzed in urinary sediment cells. Positive reactions were found in ten patients out of 48 renal transplants recipients. Of note, two patients with deteriorating renal function immediately after renal transplantation from a HCMV-seropositive organ donor initially had a HCMV-positive urine sediment that disappeared when renal function improved. This is consistent with recent data that reperfusion injury itself can reactivate latent infections [18]. In addition, immunofluorescence staining for HCMV-IEA of urine sediment in RTX patients with urinary HCMV transcripts showed positive expression in tubular epithelial cells with morphological features of membrane damage.

Of note, two non-transplant patients who were on immunosuppressive therapy also tested positive for HCMV copies in the urine sediment analysis.

Although it has been shown that HCMV infections cause cellular and humoral immune responses such as antibody production [19] and T-cell immunity [20], some of the HCMV-specific antibodies are neutralizing. It is therefore conceivable that the immune system may keep the infection at a low rate of spread. Following recovery from the disease, replication of the pathogenic virus is most likely suppressed and placed into a latent status.

Our observation of HCMV detection after transplantation in the urine sediment is in accordance with findings in animal experiments. Reactivation of latent HCMV was recently described in a murine model facilitated by implantation of a latently infected allogeneic kidney together with administration of immunosuppression. HCMV was reactivated within the allogeneic kidney and also spread to other organs [18].

This study cannot answer the question whether RT-qPCR of urine sediment cells can detect HCMV infection with renal involvement in all cases. However, the study clearly shows that immunosuppression in general and immunosuppressive therapy regimens in particular predispose to HCMV detection in urinary sediment. This is consistent with studies showing that the type of immunosuppression has a significant impact on replication of latent viruses [21]. The major limitation of the present work is the retrospective design. In addition, no follow-up urine samples were systematically collected. The HCMV DNA in plasma was detected in only a few patients and was not measured in urine as the focus was on determining the HCMV transcript UL132 in urinary cell sediment by reverse transcription of urinary cell-derived RNA. Furthermore, histologic reports from renal biopsies were not available for the entire study cohort. On the other hand, our study was strengthened by a substantial sample size and a well-characterized patient cohort consisting of immunosuppressed and non-immunosuppressed patients with acute kidney injury.

## Conclusions

This study describes a novel method for detecting HCMV infections in renal allograft patients using a noninvasive test in urine sediment. However, two non-transplant patients under immunosuppression due to an underlying disease also tested positive for HCMV. Hence, positive urinary sediment for HCMV transcripts is accompanied with immunosuppression. It does not, however, allow conclusions whether all HCMV infections can be detected or only those with viral replication.

## Data Availability

Data supporting the findings of this study are available upon request from the corresponding author.

## Abbreviations

AKI: Acute kidney injury
ATG: Antithymocyte globulin
CKD: Chronic kidney disease
D: Donor IgG status of HCMV
DGF: Delayed graft function
DNA: Deoxyribonucleic acid
dNTP: Deoxyribonucleotide triphosphate
DTT: Dithiothreitol
HCMV: Human cytomegalovirus
HCMV-IEA: HCMV immediate early antigen
IAS: Immunoadsorption
IQR: Interquartile range
IRI: Ischemia reperfusion injury
nRTX: Non-transplant recipient
PBS: Phosphate buffered saline
R: Recipient IgG status of HCMV
RNA: Ribonucleic acid
RTX: Renal transplant recipient
RPM: Revolutions per minute
RT-qPCR: Real-time quantitative polymerase chain reaction
